# The Acceptance Behavior of Smart Home Health Care Services in South Korea: An Integrated Model of UTAUT and TTF

**DOI:** 10.3390/ijerph192013279

**Published:** 2022-10-14

**Authors:** Hyo-Jin Kang, Jieun Han, Gyu Hyun Kwon

**Affiliations:** 1 Department of Service Design Engineering, Sungshin Women’s University, Seoul 02844, Korea; 2 Graduate School of Technology and Innovation Management, Hanyang University, Seoul 04763, Korea

**Keywords:** smart home health care, technology acceptance, unified theory of acceptance and use of technology (UTAUT), task technology fit (TTF), partial least square structural equation modeling

## Abstract

With the COVID-19 pandemic, the importance of home health care to manage and monitor one’s health status in a home environment became more crucial than ever. This change raised the need for smart home health care services (SHHSs) and their extension to everyday life. However, the factors influencing the acceptance behavior of SHHSs have been inadequately investigated and failed to address why users have the intention to use and adopt the services. This study aimed to analyze the influential factors and measure the behavioral acceptance of SHHSs in South Korea. This study adopted the integrated model of the unified theory of acceptance and use of technology (UTAUT) and task–technology fit (TTF) to understand the behavioral acceptance of SHHSs from users’ perceptions and task–technology fit. Multiple-item scales were established based on validated previous measurement scales and adjusted in accordance with SHHS context. Data from 487 valid samples were analyzed statistically, applying partial least square structural equation modeling. The results indicated that the integrated acceptance model explained 55.2% of the variance in behavioral intention, 44.9% of adoption, and 62.5% of the continuous intention to use SHHSs, supporting 11 of the 13 proposed hypotheses. Behavioral intention was positively influenced by users’ perceptions on performance expectancy, effort expectancy, social influence, and functional conditions. Task–technology fit significantly influenced performance expectancy and behavioral intention, validating the linkage between the two models. Meanwhile, task characteristics were insignificant to determine task–technology fit, which might stem from complex home health care needs due to the COVID-19 pandemic, but were not sufficiently resolved by current service technologies. The findings implied that the acceptance of SHHSs needs to be evaluated according to both the user perceptions of technologies and the matching fit of task and technology. Theoretically, this study supports the applicability of the integrated model of UTAUT and TTF to the domain of SHHS, and newly proposed the measurement items of TTF reflecting the domain specificity of SHHS, providing empirical evidence during the pandemic era in South Korea. Practically, the results could suggest to the planners and strategists of home health care services how to promote SHHS in one’s health management.

## 1. Introduction

With the fourth industrial revolution and information and communication technology (ICT) advancement, a highly intelligent home environment is being realized [1]. Along with the industrial investments into smart home sectors, academic research has also investigated smart homes in terms of technological capabilities, their implications on various services, and the benefits to users’ lives [2]. Technology-wise, smart homes are characterized by integrating technologies such as home automation, automatic control systems, communication networks, connection devices and services, remote access and control, and home intelligence [3,4,5]. Service-wise, smart homes provide diverse services such as management, control, monitoring, and responsive assistance through technical management of home environment [2]. Hence, the benefits that users achieve from smart homes are enhanced security and safety, convenience and efficiency, comfort and healthcare, communication and entertainment, and sustainability; consequently, they can experience a better quality of home life [2,3,4,5,6].

Particularly, smart homes have been considered to effectively support the aging population and those with chronic diseases [7,8,9,10]. The health-related advantages of smart home technologies can be summarized as operational efficiency, monitoring and management, and consultancy [2,11]. The operational efficiency of smart homes can ensure care accessibility and availability, safety, security, and comfort [8,11,12]. Real-time and long-term monitoring and disease management technologies can enable the detection of users’ health emergencies and proactive actions toward them [13,14]. The consultancy functions can promote virtual and remote medical consultations instead of physical visits to hospitals [2,11]. Therefore, smart homes can be an effective venue for continuous and non-intrusive health monitoring and the prevention of disease, while ensuring users’ quality of life and independence [14,15].

Founded on those prior studies of smart homes and their implications on health care sectors, this research inherited the term “smart home health care services (SHHSs)” and the following operationalized definition from our previous research [14,15,16], which embraces both technical and experiential perspectives:

“*Smart home health care is a health care service in one’s residence incorporated with IoT technology and ubiquitous computing, which has the characteristics of home automation and home intelligence, communication networks, and remote access and control by authorized health care personnel. It offers informal health care services such as real-time or long-term health monitoring, unobtrusive activity support without interference with daily lives, and disease prevention through anomaly detection. It can reduce care costs, allow satisfactory service experience in a comfortable and private home environment, and ensure the independence of residents [15,16]*”.

Meanwhile, a large part of previous studies on smart homes has confined their research scope to the technological perspective, such as the functions of devices, the development of infrastructure and architecture, and their applications [2,3,16,17]. In addition, some scholars pointed out that the prevailing technological focus on smart home research may imply their low acceptance in the market [2,12]. While most studies underscored potential benefits and challenges of smart home technologies, research on the empirical evidence of users’ perception and acceptance of those benefits has not been sufficiently conducted [2]. Likewise, several literature reviews on SHHSs asserted that much of the research had investigated the technological perspective including system and service development, communication infrastructure, or algorithm models, but the users’ perspectives, such as their experience and acceptance of SHHSs, have not been explored sufficiently [13,14,15].

Moreover, the COVID-19 pandemic has caused enormous changes and restrictions in the human living environment regarding work and home-life situations associated with digital transformation [18,19,20]. During lockdowns against the pandemic, digital communication technologies and infrastructure have accelerated remote and asynchronous ways of work and home-life [20]. Above all, home health care to manage and monitor one’s health status in a private and safe in-home environment has become more important than ever [21,22]. Along with technological advancement, this change brings about a rapid increase in the need for high-tech-based SHHSs and their expansion into everyday lives.

To sum up, the necessity of this research was established from (1) the increased need for home health care since the COVID-19 pandemic, and (2) the market expansion of SHHSs associated with rapid technological development, and (3) the continued lack of empirical evidence of user acceptance of SHHSs. Namely, it is necessary to investigate users’ attitudes and technology acceptance of SHHSs in the pandemic era. With these backgrounds, this study aims to analyze the user acceptance of SHHSs particularly in the South Korean context, investigating the influential factors for users’ acceptance behaviors of SHHSs, and to examine the relationships of external constructs influencing users’ intention to use and adoption.

Several studies have investigated user acceptance of home health care services such as mobile health applications and devices in Europe or China [23,24,25,26,27]. However, the primary target of those studies was mobile application services, and research on health care particularly focused on smart home environments was not sufficiently conducted. Therefore, among the various versions of technology acceptance models, this study adopts an appropriate model for technology-intensive SHHSs and establishes the measurement items reflecting the domain specificity of SHHSs. Consequently, the findings of this research could contribute to providing empirical evidence and offering additional insights into the technology acceptance of SHHSs specified in the South Korean context at the time of the pandemic era.

## 2. Theoretical Framework and Research Hypotheses

To assess the acceptance of technologies, researchers have established several models such as the technology acceptance model (TAM) [28], its well-known extensions of TAM 2 [29], and the unified theory of acceptance and use of technology (UTAUT) [30]. Notably, Venkatesh et al. proposed the UTAUT model that integrated eight models and theories of prior studies (i.e., the theory of reasoned action (TRA), TAM, the motivation model, the theory of planned behavior (TPB), a combined model of TAM and TPB, the innovation diffusion theory, the model of personal computer utilization, and the social cognitive theory) [30]. Therefore, UTAUT has been considered to have a comprehensive understanding of the acceptance procedure and a robust predictive power compared with other models [31]. It presents four determinants of behavioral intention: (1) performance expectancy (PE, adapted from the perceived usefulness of TAM), (2) effort expectancy (EE, adapted from the perceived ease of use of TAM), (3) social influence (SI, adapted from the subjective norm of TRA), and (4) facilitating conditions (FC, adapted from the perceived behavior control of TPB) [32]. Many researchers have employed the UTAUT to explain the technology acceptance behavior across diverse domains, including information technologies [24,32,33] and medical informatics [34,35].

Meanwhile, Goodhue and Thomson suggested the task technology fit (TTF) model comprehends the association between information system and individual performance; this model illustrates how the fit between technologies and users’ tasks influences individual performance in information technology systems [36]. The task–technology fit is the level to which the technology features or functions match the task requirements, and both task characteristics (TAC) and technology characteristics (TEC) are the determinants of task–technology fit [36,37,38]. In other words, when the task requirements exceed the capabilities of technologies, or when technology features demonstrate unsatisfactory performances to complete the task, the task–technology fit would decline [24]. The TTF model has been widely applied to various contexts of information systems, including online services [39,40] and mobile technologies [41,42].

Although the UTAUT has been evaluated in diverse industry contexts and can describe around 70% of the users’ usage intention [30], some limitations still exist. Even if a user considers specific information systems useful and easy to use, (s)he will not use the system when it cannot fit requirements or improve performance [43]. While the UTAUT underscores a user’s perceptions of technology, the TTF elucidates a user’s acceptance from the perspective of task–technology fit. In that sense, TTF can compensate for the limitation of UTAUT by explaining the fit between the task requirements and technical characteristics. Accordingly, several studies have combined the UTAUT and TTF models to explain user adoption behavior of advanced technology services such as mobile banking, online education, and wearable device services [24,33,43,44]. In that research, the factors of both TTF and UTAUT models significantly influenced the user’s adoption behavior [43].

As mentioned earlier, SHHS is a technology-intensive service incorporating diverse home intelligence technologies to support real-time and long-term health monitoring, unobtrusive activity support, and disease prevention [14,15]. Thus, users’ perceptions of the smart home and health care technologies are basically influential to their adoption behaviors. However, whether the technologies of SHHSs can satisfy users’ requirements and support their tasks sufficiently would be at a different level to the perception of technologies. In other words, the acceptance of SHHSs needs to be approached considering both the technical complexity of smart homes and the task pertinence of home healthcare. Therefore, to establish a comprehensive understanding of the users’ adoption behavior of SHHSs, embracing the TTF perspective as well as the typical UTAUT model is critical.

**Figure 1:**
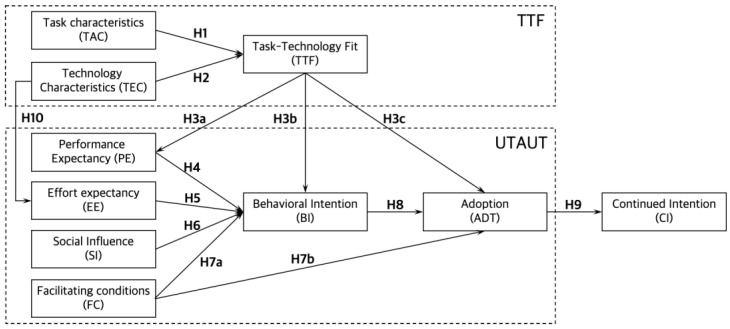
Theoretical framework (TTF: task technology fit, UTAUT: unified theory of acceptance and use of technology).

Accordingly, this study adopted the integrated model of UTAUT and TTF from previous research [24,35,43,44]. The integrated research framework was established as illustrated in Figure 1, and the hypotheses are listed in Table 1.

The proposed model consists of ten constructs contextualized in the SHHS context. We included behavioral intention (BI), adoption (ADT), and continued intention (CI) to measure the progress of user acceptance as they are widely adopted predictors of the actual acceptance of technology-intensive services [24,33,43,44,45,46].

The influence of TAC on TTF (H1) and that of TEC on TTF (H2) were adopted from the TTF model [36,37]. The influences on BI from PE (H4), from EE (H5), from SI (H6), and from FC (H7a) were adopted from the UTAUT model [30].

The relationship between the two models was hypothesized by referring to previous studies. Several scholars [24,33,43,44] have reported that TTF positively influences PE (H3a), BI (H3b), and ADT (H3c). Likewise, in the context of SHHSs, users can perceive that a SHHS could enhance their home health care performance only when the functions of SHHS can match well with home health care tasks (i.e., task–technology fit). Moreover, the characteristics of SHHSs (e.g., ubiquitous and real-time services) can support users’ continuous health monitoring and thus diminish their effort cost. Based on this, the hypothesis that the technology characteristics of SHHSs positively affect effort expectancy (H10) was settled [24,33].

Meanwhile, in the original UTAUT model [30], demographic variables such as gender, age, experience, and voluntariness of use were included as moderators. However, due to the complexity of the unified model with many constructs, most of the previous studies adopting the unified model of UTAUT and TTF focused on the link between two models without moderators to explore the validity and applicability of the unified model [24,33,43,44]. Likewise, this study also aims to test the applicability of the unified model in the SHHS domain, thus any moderating variable was not considered in this research framework.

## 3. Materials and Methods

### 3.1. Measurement

Multiple-item scales were established to measure the constructs. Measurement items were adopted from pertinent previous studies and adjusted to be suitable for the SHHS context, as shown in Table 2. Keywords to represent the meaning of each measurement item were defined as properties. Notably, the properties of TAC and TEC were developed from literature to delineate the characteristics of SHHSs comprehensively. The tasks of SHHSs embrace the needs of real-time (TAC1) and long-term health monitoring (TAC2), disease prevention by detecting anomalies (TAC3), emergency management (TAC4), unobtrusive activity assistance (TAC5) [13,14], continuous customization in adaptive systems (TAC6), connection with diversified stakeholders (TAC7) [47,48], and finally improved privacy and life independence (TAC8) [49]. The technology characteristics for SHHSs include the technological requirements of smart homes such as ubiquitous (TEC1), real-time (TEC2), and reliable services (TEC3) [24,33], home intelligence (TEC4), home automation (TEC5), data communication based on IoT technologies (TEC6), and remote control and access (TEC7) [3,50,51,52,53]. The properties of task–technology fit involve the sufficiency and appropriateness of SHHS functions and the fulfillment of user needs, function-wise and quality-wise [24,33,43,45,54].

The measurement items of UTAUT model constructs (i.e., PE, EE, SI, FC, BI, ADT, and CI) were adapted from the validated scales in the relevant studies [24,30,33,36,43,44,55,56]. All the items were measured with an 11-point Likert scale (0, strongly disagree; 5, indecisive; 10, strongly agree). Some research in social studies recommended an 11-point scale, as it enhanced scale sensitivity, normality, and understandability [57,58]. Accordingly, this study also adopted such a scale to capture the level of user perception in fine granularity.

**Table 2:** Measurement items and the sources of constructs.

Construct	Items	Properties	Measures	References
Task characteristics (TAC)	TAC1	Real-time health monitoring	In my home environment, I need to monitor my health status in real-time.	[13,14]
	TAC2	Long-term health monitoring	In my home environment, I need to monitor my health status in the long term.	
	TAC3	Disease prevention by detecting anomalies	In my home environment, I need to prevent disease by detecting anomalies in my health status.	
	TAC4	Emergency management	In my home environment, I need to deal with emergencies by detecting anomalies in my health status.	
	TAC5	Unobtrusive activity assistance	In my home environment, I need services to assist my health care activities unobtrusively.	
	TAC6	Continuous customization (adaptive system)	In my home environment, I need services to be continuously customized according to my health status.	[47,48]
	TAC7	Connection with diversified stakeholders	In my home environment, I need to be connected to diverse stakeholders who will support my health care.	
	TAC8	Improved privacy and independence	I need to improve privacy and life’s independence through home health care.	[49]
Technology characteristics (TEC)	TEC1	Ubiquitous service	Smart home health care technologies provide ubiquitous health care services.	[24,33]
	TEC2	Real-time service	Smart home health care technologies provide real-time health care services.	
	TEC3	Reliable service/security	Smart home health care technologies provide reliable health care services.	
	TEC4	Home intelligence	Smart home health care technologies provide intelligent health care services.	[3,50,51,52,53]
	TEC5	Home automation	Smart home health care technologies provide automated health care services.	
	TEC6	Communication network & IoT technology	Smart home health care technologies provide health care data and information based on the communication network and IoT technologies.	
	TEC7	Remote control and access	Smart home health care technologies provide health care services with remote control and access through various smart devices.	
Task–technology Fit (TTF)	TTF1	Sufficiency	In my health care, the functions of SHHSs are sufficient (enough).	[24,33,43,45,54]
	TTF2	Appropriateness	In my health care, the functions of SHHSs are appropriate.	
	TTF3	Meeting needs of function	The functions of SHHSs fully meet my health care requirements.	
	TTF4	Meeting needs of quality	The quality of SHHSs can fully meet my health care requirements.	
Performance expectancy (PE)	PE1	Perceived usefulness	I feel SHHSs are useful in my health care.	[24,30,33,43,56]
	PE2	Extrinsic motivation	Using SHHSs accelerates the process of health care service provision.	
	PE3	Relative advantage	Using SHHSs increases my chances of managing my health.	
	PE4	Outcome expectation	Using SHHSs enables me to ameliorate my health status.	
Effort expectancy	EE1	Perceived ease of use	Skillfully using SHHSs is easy for me.	
	EE2	Complexity	I find SHHSs difficult to use. (reverse)	
	EE3	Learnability	Learning how to use SHHSs is easy for me.	
	EE4	Understandability	My interactions with SHHSs are clear and understandable.	
Social influence	SI1	Subjective norm	People who are important to me think that I should use SHHSs.	
	SI2	Social factors	People who influence my behavior think that I should use SHHSs.	
	SI3	Image	I find that using SHHSs is a fashionable and popular way of health care.	
Facilitating conditions	FC1	Perceived behavioral control	I have the necessary resources (system, tools, circumstances) to use SHHSs.	
	FC2	Perceived behavioral control	I have the necessary knowledge to use SHHSs.	
	FC3	Facilitating conditions	If I have difficulty using SHHSs, there will be support from the system to help me.	
	FC4	Compatibility	SHHSs are compatible with other technologies or systems I use.	
Behavioral intention	BI1	Consideration	I will consider using SHHSs for my health care.	[24,30,44,56]
	BI2	Intention	I have the intention of using SHHSs for my health care.	
	BI3	Want	I would like to use SHHSs for my health care if I have an opportunity.	
	BI4	Plan	I have a plan to use SHHSs for my health care.	
Adoption	ADT1		I use SHHSs to monitor my health status.	[30,33,36,44]
	ADT2		I use SHHSs to prevent disease and to deal with emergencies.	
	ADT3		I use SHHSs for customized health care.	
	ADT4		I use SHHSs for my overall health care.	
Continued intention	CI1		I will continue using SHHSs to monitor my health status.	[40,43,45,46,55]
	CI2		I will continue using SHHSs to prevent disease and to deal with emergencies.	
	CI3		I will continue using SHHSs for continuously customized health care.	
	CI4		I can develop a habit of using SHHSs regularly.	

### 3.2. Data Collection

Screening questions were introduced at the beginning of the survey, asking about users’ experiences relevant to SHHSs. Five categories (personal health care devices, wearable devices, health information app services, customized health care app services, and telemedicine services [59]) were represented, together with each one’s sample products or services. A user who did not check any experience with the suggested SHHSs was automatically dropped from the survey. Demographic questions followed at the end of the survey including gender, age, residential district, marital status, and household members. Moreover, two redundant but reverse-coded questions were added in the middle of the measurement questionnaire (TTF5 and BI5) to exclude inattentive respondents.

As sampling methods, stratified sampling for gender (i.e., the same quota for men and women) and random sampling techniques were adopted. Data collection was conducted with an online survey method through a web-based platform. The survey was randomly distributed by Macromill Embrain Inc. (one of the biggest online survey companies in South Korea) using the company’s respondent panels. The response data were collected between 1 February 2021 and 5 February 2021. Inattentive responses were eliminated at the data collection stage by the company according to the reverse-coded questions. The initial responses totalled 503; then, eight outliers were removed according to a normality test. Probably due to the web survey method, the number of participants aged in their 20s and 30s took up a large share (77.2%), and the number of those aged 60 or over was deficient (5 in their 60s and 3 in their 70s). So, those in their 60s and older were excluded because of insufficient samples to represent that age group. Finally, the data for analysis was reduced to 487. Table 3 represents the demographic characteristics of participants.

### 3.3. Statistical Procedure

Data were analyzed using SPSS (version 25, IBM Corp., Armonk, NY, USA) and SmartPLS (version 3, SmartPLS GmbH, Oststeinbek Germany) software. This study adopted the partial least square structural equation modeling (PLS-SEM) technique to examine the theoretical framework and test the ten hypotheses. Generally, covariance-based SEM is selected when a research goal is theory testing and confirmation or comparison of alternative theories [60]. Meanwhile, the partial least square method, a variance-based SEM, is a good alternative to covariance-based SEM in cases of studies in the early stage of theory building or an extension of an existing structural theory [60,61,62,63]. Moreover, the PLS methods are especially convenient for large and complex research models to test both formative and reflective constructs compared to covariance-based SEM methods [26,62]. Therefore, the PLS technique was appropriate for our research context because the SHHSs are still under-explored from the perspective of task–technology fit and technology acceptance. Moreover, the combination of UTAUT and TTF models generated a complex research framework with many constructs. For these reasons, most of the previous studies introducing the unified model of UTAUT and TTF adopted the PLS-SEM technique due to the complexity of the research model [24,33,44,56,64], so this study also followed the proven method appropriate for this unified model.

Above all, descriptive statistics were conducted on the measurement items. Next, the model was evaluated in two stages: measurement model (i.e., outer model) and structural model (i.e., inner model) evaluation. The measurement model was evaluated by (1) internal consistency reliability, (2) convergent validity, and (3) discriminant validity of measurements [65,66]. Then, the structural model was evaluated by (1) multicollinearity, (2) effect size (*f*^2^), (3) coefficient of determination (*R*^2^), (4) predictive relevance (*Q*^2^), and (5) path coefficients and their significance [65,66].

Lastly, path coefficients and their significance were estimated by bootstrapping 5000 subsamples [61,67]. Resampling techniques, such as bootstrapping, are necessary to gain the parameters’ standard errors because PLS does not assume a particular data distribution [66], and the resample size of 5000 is most frequently adopted for the bootstrapping setting [61]. Finally, path coefficient, *t* values, and *p* values were examined to distinguish the relationships between constructs in the structural model. Accordingly, the hypotheses were statistically proven with *t* values greater than 1.96 and *p* values lower than 0.01.

## 4. Results

### 4.1. Descriptive Statistics

Table 4 demonstrates descriptive statistics of the measurement items. The mean values ranged from 5.21 (ADT2) to 7.27 (BI3). Overall, TTF and ADT showed a relatively low level of mean values, and TEC and BI included the variables with comparatively high mean values. All measurement items followed a normal distribution with all their absolute values of skewness and kurtosis indicating less than 1.

### 4.2. Measurement Model Evaluation

The measurement model was evaluated by (1) internal consistency reliability, (2) convergent validity, and (3) discriminant validity of measurements [65,66].

First, internal consistency reliability requires Cronbach’s α, Dijkstra–Henseler’s rho, and composite reliability with each value larger than 0.70 for good reliability [68,69]. Table 5 exhibits the analysis of the measurement model. In terms of internal consistency, all values of Cronbach’s α, Dijkstra–Henseler’s rho, and composite reliability for each construct were larger than 0.70, ensuring good reliability.

Second, convergent validity is determined satisfactory when the outer loading value of each measurement item exceeds 0.70, indicator reliability is greater than 0.50, and the average variance extracted (AVE) of each construct surpasses 0.50 [60,67]. For this model, as shown in Table 5, the outer loading value of each item surpassed 0.70; the indicator reliability of each item was greater than 0.50; the AVE of each construct exceeded 0.50.

Third, discriminant validity is examined using the Fornell–Larcker criterion when the square root of AVE is greater than the highest value among the correlations between construct items [70]. Discriminant validity is also acceptable when the outer loading of each measurement item is higher on its corresponding construct than the cross-loadings on other constructs [67]. Discriminant validity was determined satisfactory using the Fornell–Larcker criterion (Table A1); the square root of AVE of each construct was larger than its correlation coefficients with other constructs. Discriminant validity was also verified as acceptable since the outer loading of each item was higher on its corresponding construct than the cross-loadings on other constructs (Table A2). Accordingly, the measurement model passed the tests of consistency and validity with satisfactory results.

### 4.3. Structural Model Evaluation

The structural model was evaluated by (1) multicollinearity, (2) effect size (*f*^2^), (3) coefficient of determination (*R*^2^), (4) predictive relevance (*Q*^2^), and (5) path coefficients and their significance [65,66].

First, the model is assessed as acceptable without multicollinearity when the variance inflation factor (VIF) between constructs is less than 5 [60]. Multicollinearity of this model was tested with inner variance inflation factor (VIF) values between constructs, as shown in Table 6. All VIF values were less than 5, so the structural model was acceptable without multicollinearity.

Second, the effect size between constructs means the relative impact of exogenous variables on endogenous variables. It is assessed as low-level with the *f*^2^ value around 0.02, middle-level with its value around 0.15, and high-level with its value around 0.35 [65]. Table 6 shows the effect size (*f*^2^) between constructs of this structural model. The value between TAC and TTF and that between TTF and ADT were assessed insufficient with the values under 0.02, while other values satisfied more than the low-level effect size.

Third, the coefficient of determination (*R*^2^) values of endogenous variables show low-level explanation with its value around 0.25, middle-level with its value around 0.5, and high-level with its value over 0.75. In this model, *R*^2^ values of endogenous variables showed an acceptable level except EE (TTF: *R*^2^ = 0.263; PE: *R*^2^ = 0.433; EE: *R*^2^ = 0.193; BI: *R*^2^ = 0.552; ADT: *R*^2^ = 0.449; CI: *R*^2^ = 0.625).

Fourth, the predictive relevance of the PLS-SEM model is evaluated by Stone–Geisser’s *Q*^2^ value; when its values of endogenous variables are over 0, the model is considered to have predictive relevance on those variables [71]. In this model, *Q*^2^ values of all endogenous variables represented an acceptable level (i.e., *Q*^2^ > 0) (TTF: *Q*^2^ = 0.221; PE: *Q*^2^ = 0.340; EE: *Q*^2^ = 0.142; BI: *Q*^2^ = 0.497; ADT: *Q*^2^ = 0.398; CI: *Q*^2^= 0.544). Accordingly, the structural model was generally evaluated acceptable to determine pass coefficients and to test hypotheses.

### 4.4. Hypothesis Testing

**Figure 2:**
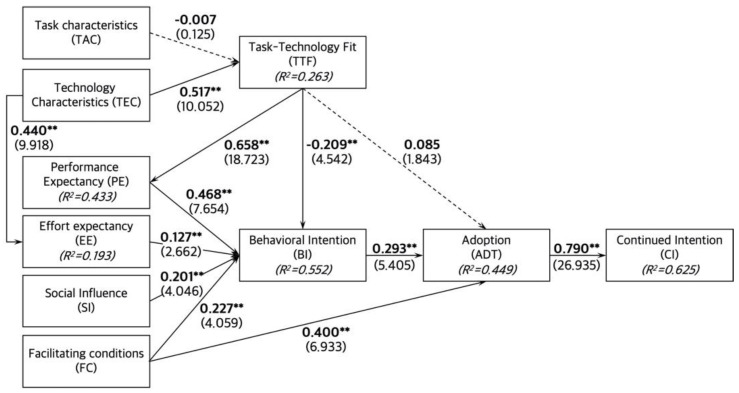
Structural model evaluation (** *p* < 0.01).

The results of hypothesis testing with path coefficients are exhibited in Table 6 and depicted in Figure 2. Task characteristics of SHHSs exerted a slightly negative impact on the task–technology fit, but H1 was not supported with insufficient significance. Technology characteristics of SHHSs had a positive influence on the task–technology fit and supported H2. Task–technology fit of SHHSs directly affected performance expectancy and behavioral intention, thus H3a and H3b were supported. However, the impact of task–technology fit on the adoption of SHHSs was not significant, and H3c was rejected. Performance expectancy, effort effectiveness, social influence, and facilitating conditions had a direct impact on the behavioral intention to use SHHSs, supporting H4, H5, H6, and H7a. The positive impacts of facilitating conditions and behavioral intention on the adoption of SHHSs were significant, thus H7b and H8 were supported. The adoption positively affected the continued intention to use SHHSs, supporting H9. Lastly, technology characteristics of SHHSs positively affected effort expectancy, and H10 was supported.

## 5. Discussion

### 5.1. Principal Findings

Concerning the TTF model, the insignificant and negative relationship between TAC and TTF (H1, *β* = −0.007) and the positive relationship between TEC and TTF (H2, *β* = 0.517 **) are consistent with previous research in the case of MOOC service acceptance [43]. In several studies on the adoption of mobile banking or wearable devices, the task properties showed a lower significance level of impact on TTF than technology [24,44]. Other studies presented a significant but negative relationship between TAC and TTF [36,72]. Those researches interpreted that TTF decreases as task requirements increase; tasks can become too complex and extensive for technology to support the appropriate level of performance; if technology functionality increases enough to support the task expectations, the TTF increases [36,72]. Likewise, home health care tasks can become too diverse and complex, particularly due to the COVID-19 pandemic. Task characteristics such as real-time and continuous health monitoring and emergency management are critical issues in self-quarantine systems. Meanwhile, SHHS technologies, despite their current advancement, might not sufficiently meet users’ expected performance levels. This is also supported by the result that the average value of all TTF measurement items (5.66) was lower than that of TAC (6.47) and TEC (6.80).

Regarding the UTAUT model, the analysis results proved that PE, EE, SI and FC significantly affected BI, explaining 55.2% of BI variances, consistent with the previous evidence of UTAUT studies [24,30,33,43,73]. Remarkably, the most influential factor that affects users’ behavioral intention is PE (H4, *β* = 0.468 **). When SHHS functions meet the users’ expected level of performance (i.e., usefulness, relative advantages, and outcome expectation), users’ intention to use SHHSs can be influenced. Meanwhile, EE had a relatively low impact on the intention of SHHS acceptance (H5, *β* = 0.127 **). The reason might be that users have become familiar with using high-tech services, and they may think that adopting SHHS does not require much effort [24]. Additionally, they might accept more effort-cost when SHHS technologies offer expected functions (H10, *β* = 0.440 **) [40]. The influence patterns of high PE and low EE are also supported by previous studies in the cases of mobile banking [33] and health care wearable devices [24]. Moreover, FC such as the technological infrastructure to ensure a seamless experience of SHHSs (e.g., compatible tools and system circumstances) positively leads to BI (H7a, *β* = 0.227 **) and the adoption (H7b, *β* = 0.400 **), in line with previous mobile banking cases [33,44].

The relationship between TTF and UTAUT models is primarily exhibited by the influence of TTF on PE. In this study, the TTF has a strong positive impact on PE (H3a, *β* = 0.658 **), and TTF explains 43.3% of PE variation; it is also congruent with similar studies’ results [33,43,44]. Hence, the fit between users’ requirements on home health care and SHHS technologies would directly influence users’ expectations of home health care performance. The higher the TTF level is, the more useful the users perceive the SHHS.

Meanwhile, the linkage between the two models is also presented by the influence of TTF on BI and ADT. TTF had a significant but negative effect on the intention to use SHHSs (H3b, *β* = −0.209 **), and it had an insignificant impact on the adoption of SHHSs (H3c, *β* = 0.085). Users might agree that smart home technologies for home health care are highly developed, and the requirements for home health care services have become diverse and complex. However, the public might consider that those technologies have not been sufficiently popularized for the home health care services to address their needs and to be adopted. This is also supported by Gartner’s hype cycle for emerging technologies in 2018; SHHS relevant technologies (i.e., connected home, IoT platform, and blockchain for data security) was anticipated to reach a mainstream market in five to ten years [74]. So, the insufficient TTF could lead to a negative influence on BI or an insignificant impact on the adoption of SHHS. In a similar instance, the previous two studies on mobile banking adoption, which integrated UTAUT and TTF models, were conducted in 2010 [33] and 2014 [44]. Though the mobile banking technology developed further in 2014, the significant influence of TTF on the adoption in the former was changed as insignificant in the latter. It might be considered that the maturity and popularization of the market service have not been sufficiently achieved compared to technological development and advanced needs.

### 5.2. Contributions and Implications

This research has both theoretical and practical implications. On a theoretical level, firstly, this study supports the applicability of the integrated model of UTAUT and TTF, which recently was explored in technology-intensive industries, to the domain of SHHS. As mentioned in the introduction, the acceptance of SHHS needs to consider both the technical complexity of smart homes and the task pertinence of home healthcare. Therefore, the integrated model of TTF and UTAUT was adapted to establish a comprehensive understanding of the users’ adoption behavior of SHHS. A limited number of previous studies combining UTAUT and TTF models engaged with mobile banking, online education, and wearable devices [24,33,43,44], and the relationships between the two models were differently examined by service. Our research model included the possible links from the previous research (i.e., TTF to PE, BI, and ADT; TEC to EE; ADT to CI) and examined their validity in the SHHS case. Hence, the findings explained behavioral acceptance from the perspective of users’ perception of task–technology fit and assured the importance of integrating TTF elements into acceptance theories when assessing determinants of user acceptance towards SHHS [24,43].

Second, this study establishes the measurement items reflecting the domain specificity of SHHS. The constructs of task characteristics and technology characteristics should reflect the specificity of relevant industry domains. This research developed the distinct measurement items for SHHS derived from the literature, thus it suggested the full questionnaire set of a unified model of UTAUT and TTF specialized for the user acceptance of SHHS. It can be utilized and adapted to other contexts of SHHS when evaluating the users’ adoption behaviors.

Third, this study provides empirical evidence for the research on user acceptance of SHHS during the pandemic era in South Korea. As noted in the introduction, much of the research on SHHS has investigated the technological perspective but lacked the users’ perspectives such as their experience and acceptance of SHHS. With the increased need for home health care since the pandemic and the market expansion of SHHSs associated with rapid technological development, research on the actual users’ acceptance was required. In that sense, this study could be meaningful in providing empirical evidence of service acceptance by collecting a large number of actual SHHS users.

On a practical level, these research findings could help home health care service planners and marketers promote SHHS in an individual’s health management. With the COVID-19 pandemic, user needs for home health care have become complicated. Despite the current rapid development of related technologies, technological maturity and service popularization do not seem to have been achieved sufficiently for users to realize. When their complex needs can be satisfied through the convergence of various technologies as well as smart home technology (TTF), users would feel that SHHS is useful and beneficial (PE) and this may finally lead to adoption. Therefore, it is recommended to communicate effectively with potential customers how convergent technologies of the service solve the specific needs of the user. Moreover, in terms of FC, strategies to improve technological infrastructure to ensure a seamless experience of SHHSs (e.g., compatible service with diverse usage contexts) would be required to promote user acceptance.

### 5.3. Limitations and Future Research

This study has several limitations. First, alternative technologies or service platforms for home health care may exist. The basic assumption of SHHS in this study is the situation in which home health care services are implemented based on smart home technology. However, users may not necessarily consider only health care services based on smart home technology to achieve the purpose of self-health management. As the needs for personal health care become diversified due to the COVID-19 situation, users could conveniently perform home health care by utilizing various means such as exercise equipment, services, and platforms. As in this study’s result, even if the need for home health care tasks is high, it may not sufficiently lead to task–technology fit or acceptance of actual SHHS. Accordingly, it would be necessary to embrace various technologies and service methods for home health care in future studies.

Second, this study was conducted in South Korea, and the research findings might be difficult to generalize due to specificity to Korean users. Generally, South Korea is often considered as a testbed of global ICT brand companies, due to highly developed information technology infrastructure, the nation’s innovativeness, and the people’s digital friendliness [75,76]. Generally, Korean users may have a high level of technological expectation, and they tend to be early adopters. Moreover, due to the data collection method of the web survey, those in their 60s and older were excluded because of insufficient samples. Hence, the respondent group of this study might have a tendency to high-level innovativeness. They might consider that the task–technology fit level is unsatisfactory to adopt SHHSs more than another country’s citizens would. Therefore, comparative studies targeting users from other countries may be required to validate the generalizability of this research model.

Third, this study did not consider any comparative study by user group or by period because the primary research aim was to investigate the applicability of the unified model in the SHHS domain. However, in terms of the user group, demographic variables such as gender, age, or the level of digital literacy can be included as moderators in the research framework. Especially, the acceptance behavior of SHHS might be influenced by the level of individual digital literacy or innovativeness considering the technology-intensive service characteristics. In terms of period, research to capture users’ attitudinal changes toward SHHS could be a meaningful opportunity to validate the unified model. The empirical survey of this study was conducted in the middle of the COVID-19 period and the results could be limited to representing the context only during the pandemic period. Therefore, further studies to compare the users’ behavioral changes between the periods before and after the pandemic would be valuable.

## 6. Conclusions

This study aimed to analyze the influential factors and measure the behavioral acceptance of SHHSs in South Korea. This study adopted the integrated model of UTAUT and TTF and contextualized it, involving SHHS characteristics to explain the behavioral acceptance of SHHSs from users’ perceptions and task–technology fit. The results indicated that our integrated acceptance model explained 55.2% of the variance in behavioral intention, 44.9% of adoption and 62.5% of the continuous intention to use SHHSs, supporting 11 of the 13 proposed hypotheses. Users’ perceptions of PE, EE, SI and FC can positively anticipate BI to accept SHHSs, having relatively strong influence from PE and FC. Furthermore, TTF has an influence on PE and BI, and indirectly on ADT and CI; it implies that the consideration of matching fit of task and technology, as well as user perceptions of technologies, is essential to evaluate the acceptance of SHHSs [34]. Meanwhile, TAC was insignificant to determine TTF, which might stem from the complicated needs of home health care due to the COVID-19 pandemic but was not sufficiently resolved by current service technologies. The findings implied that the acceptance of SHHSs needs to be evaluated with consideration of both the user perceptions on technologies and the matching fit of task and technology. Theoretically, this study supports the applicability of the integrated model of UTAUT and TTF to the domain of SHHS, and newly proposed measurement items of TTF reflecting the domain specificity of SHHS, providing empirical evidence during the pandemic era in South Korea. Practically, the results could suggest to the planners and strategists of home health care services how to promote SHHS in health management.

## Figures and Tables

**Table 1:** Hypotheses list.

Label	Hypotheses
H1	The task characteristics of SHHSs * positively affect task–technology fit.
H2	The technology characteristics of SHHSs positively affect task–technology fit.
H3a	Task–technology fit positively affects the performance expectancy of SHHSs.
H3b	Task–technology fit positively affects the behavioral intention to use SHHSs.
H3c	Task–technology fit positively affects the adoption of SHHSs.
H4	Performance expectancy positively affects the behavioral intention to use SHHSs.
H5	Effort expectancy positively affects the behavioral intention to use SHHSs.
H6	Social influence positively affects the behavioral intention to use SHHSs.
H7a	Facilitating conditions positively affect the behavioral intention to use SHHSs.
H7b	Facilitating conditions positively affect the adoption of SHHSs.
H8	Behavioral intention to use SHHSs positively affects the adoption of SHHSs.
H9	The adoption of SHHSs positively affects the continued intention to use SHHSs.
H10	The technology characteristics of SHHSs positively affect effort expectancy.

* SHHSs: smart home health care services.

**Table 3:** Participants’ demographic characteristics.

Characteristics		Values, n (%)
Gender	Men	241 (49.5)
	Women	246 (50.5)
Age group	20s	184 (37.8)
	30s	192 (39.4)
	40s	72 (14.8)
	50s	39 (8.0)
Residential district	Capital area—Seoul	160 (32.9)
	Capital area—Incheon, Gyeonggi-do	151 (31.0)
	Metropolitan cities	80 (16.4)
	Other districts	96 (19.7)
Marital status	Single	313 (64.3)
	Married	174 (35.7)
Household members	1 person	87 (17.9)
	2 people	80 (16.4)
	3 people	141 (29.0)
	4 people	135 (27.7)
	5 or more	44 (9.0)
SHHS experiences (multiple answers)	Personal health care devices	275 (56.5%)
	Wearable devices	294 (60.4%)
	Health information app services	313 (64.3%)
	Customized health care app services	334 (68.6%)
	Telemedicine services	60 (12.3%)

**Table 4:** Descriptive statistics of the measurement items.

Construct	Item	Mean (SD)	95% CI	Skewness	Kurtosis
Task characteristics (TAC)	TAC1	6.26 (2.68)	[6.02, 6.50]	−0.67	−0.01
	TAC2	6.50 (2.55)	[6.27, 6.73]	−0.76	0.35
	TAC3	7.02 (2.39)	[6.81, 7.24]	−0.88	0.77
	TAC4	6.98 (2.61)	[6.74, 7.21]	−0.88	0.31
	TAC5	6.05 (2.69)	[5.81, 6.29]	−0.52	−0.22
	TAC6	6.32 (2.64)	[6.08, 6.55]	−0.68	0.07
	TAC7	6.03 (2.62)	[5.79, 6.26]	−0.45	−0.25
	TAC8	6.61 (2.46)	[6.39, 6.83]	−0.59	0.18
Technology characteristics (TEC)	TEC1	7.02 (1.89)	[6.85, 7.19]	−0.33	0.46
	TEC2	6.87 (1.88)	[6.70, 7.03]	−0.28	0.59
	TEC3	6.57 (1.96)	[6.40, 6.75]	−0.09	0.14
	TEC4	6.72 (1.96)	[6.55, 6.90]	−0.32	0.46
	TEC5	6.66 (2.02)	[6.48, 6.83]	−0.35	0.32
	TEC6	6.80 (1.93)	[6.63, 6.97]	−0.32	0.49
	TEC7	6.97 (1.90)	[6.80, 7.14]	−0.31	0.26
Task-technology Fit (TTF)	TTF1	5.72 (2.08)	[5.53, 5.90]	0.02	0.05
	TTF2	5.94 (1.94)	[5.76, 6.11]	0.14	0.01
	TTF3	5.50 (2.07)	[5.31, 5.68]	0.05	0.12
	TTF4	5.46 (1.99)	[5.28, 5.64]	0.05	0.36
Performance expectancy (PE)	PE1	6.30 (1.86)	[6.14, 6.47]	−0.12	0.41
	PE2	6.16 (1.90)	[5.99, 6.33]	−0.15	0.76
	PE3	6.80 (1.87)	[6.63, 6.96]	−0.40	0.75
	PE4	6.79 (1.83)	[6.63, 6.96]	−0.30	0.66
Effort expectancy (EE)	EE1	6.58 (1.96)	[6.40, 6.75]	−0.20	0.03
	EE2	6.63 (1.97)	[6.46, 6.81]	−0.05	−0.36
	EE3	6.76 (1.96)	[6.59, 6.94]	−0.19	−0.08
	EE4	6.24 (1.83)	[6.08, 6.40]	0.21	−0.11
Social influence (SI)	SI1	5.72 (2.54)	[5.49, 5.94]	−0.30	−0.12
	SI2	5.56 (2.51)	[5.34, 5.79]	−0.27	−0.05
	SI3	6.64 (2.06)	[6.46, 6.83]	−0.39	0.48
Facilitating conditions (FC)	FC1	5.83 (2.25)	[5.63, 6.03]	−0.36	0.22
	FC2	6.07 (2.10)	[5.89, 6.26]	−0.40	0.49
	FC3	6.21 (1.95)	[6.03, 6.38]	−0.14	0.09
	FC4	6.14 (2.03)	[5.96, 6.32]	−0.26	0.45
Behavioral intention (BI)	BI1	7.07 (1.93)	[6.90, 7.25]	−0.27	−0.35
	BI2	7.16 (1.98)	[6.98, 7.33]	−0.50	0.10
	BI3	7.27 (2.03)	[7.09, 7.45]	−0.53	−0.09
	BI4	7.05 (2.10)	[6.86, 7.24]	−0.55	0.16
Adoption (ADT)	ADT1	5.45 (2.55)	[5.22, 5.67]	−0.50	−0.09
	ADT2	5.21 (2.62)	[4.98, 5.44]	−0.37	−0.34
	ADT3	5.62 (2.52)	[5.40, 5.84]	−0.50	0.04
	ADT4	5.73 (2.53)	[5.51, 5.96]	−0.56	0.07
Continued intention (CI)	CI1	6.41 (2.27)	[6.20, 6.61]	−0.48	0.26
	CI2	6.20 (2.38)	[5.99, 6.41]	−0.56	0.22
	CI3	6.47 (2.30)	[6.27, 6.68]	−0.57	0.31
	CI4	6.44 (2.32)	[6.23, 6.64]	−0.58	0.44

**Table 5:** The measurement model evaluation.

Construct	Item	Convergent Validity			Internal Consistency Reliability		
		Outer Loading	Indicator Reliability	AVE	Cronbach α	Rho Value	Composite Reliability
		(>0.70)	(>0.50)	(>0.50)	(>0.70)	(>0.70)	(>0.70)
Task characteristics (TAC)	TAC1	0.839	0.703	0.733	0.948	0.952	0.956
	TAC2	0.870	0.757				
	TAC3	0.870	0.758				
	TAC4	0.836	0.699				
	TAC5	0.859	0.738				
	TAC6	0.879	0.772				
	TAC7	0.887	0.787				
	TAC8	0.807	0.652				
Technology characteristics (TEC)	TEC1	0.861	0.741	0.795	0.957	0.958	0.964
	TEC2	0.903	0.815				
	TEC3	0.882	0.779				
	TEC4	0.912	0.832				
	TEC5	0.900	0.810				
	TEC6	0.890	0.792				
	TEC7	0.892	0.795				
Task–technology Fit (TTF)	TTF1	0.903	0.816	0.852	0.942	0.944	0.958
	TTF2	0.925	0.856				
	TTF3	0.938	0.880				
	TTF4	0.924	0.854				
Performance expectancy (PE)	PE1	0.885	0.783	0.803	0.918	0.920	0.942
	PE2	0.884	0.781				
	PE3	0.923	0.851				
	PE4	0.893	0.797				
Effort expectancy (EE)	EE1	0.924	0.853	0.771	0.901	0.930	0.931
	EE2	0.779	0.607				
	EE3	0.923	0.851				
	EE4	0.879	0.773				
Social influence (SI)	SI1	0.875	0.766	0.754	0.845	0.889	0.902
	SI2	0.878	0.771				
	SI3	0.852	0.725				
Facilitating conditions (FC)	FC1	0.867	0.751	0.765	0.897	0.898	0.929
	FC2	0.897	0.804				
	FC3	0.854	0.730				
	FC4	0.880	0.775				
Behavioral intention (BI)	BI1	0.949	0.901	0.912	0.968	0.968	0.976
	BI2	0.974	0.948				
	BI3	0.950	0.903				
	BI4	0.946	0.894				
Adoption (ADT)	ADT1	0.953	0.908	0.898	0.962	0.964	0.972
	ADT2	0.917	0.841				
	ADT3	0.964	0.929				
	ADT4	0.956	0.914				
Continued intention (CI)	CI1	0.956	0.913	0.879	0.954	0.955	0.967
	CI2	0.908	0.825				
	CI3	0.965	0.931				
	CI4	0.920	0.846				

**Table 6:** Structural model evaluation and hypothesis test results.

Hypothesis (Path)	VIF	*f* ^2^	Path Coefficient (*β*)	*t* Value	*p* Value	Support
H1 (TAC → TTF)	1.504	0.000	−0.007	0.125	0.900	No
H2 (TEC → TTF)	1.504	0.241	0.517	10.052	0.000	Yes
H3a (TTF → PE)	1.000	0.764	0.658	18.723	0.000	Yes
H3b (TTF → BI)	1.875	0.052	−0.209	4.542	0.000	Yes
H3c (TTF → ADT)	1.440	0.009	0.085	1.843	0.065	No
H4 (PE → BI)	2.664	0.183	0.468	7.654	0.000	Yes
H5 (EE → BI)	1.721	0.021	0.127	2.662	0.008	Yes
H6 (SI → BI)	1.923	0.047	0.201	4.046	0.000	Yes
H7a (FC → BI)	2.227	0.052	0.227	4.059	0.000	Yes
H7b (FC → ADT)	1.906	0.152	0.400	6.933	0.000	Yes
H8 (BI → ADT)	1.553	0.101	0.293	5.405	0.000	Yes
H9 (ADT → CI)	1.000	1.666	0.790	26.935	0.000	Yes
H10 (TEC → EE)	1.000	0.239	0.440	9.918	0.000	Yes

## Data Availability

Not applicable.

## Appendix A

**Table A1:** The square root of AVEs (in bold) and correlation coefficients.

	TAC	TEC	TTF	PE	EE	SI	FC	BI	ADT	CI
TAC	**0.856**	-	-	-	-	-	-	-	-	-
TEC	0.579	**0.892**	-	-	-	-	-	-	-	-
TTF	0.292	0.513	**0.923**	-	-	-	-	-	-	-
PE	0.534	0.720	0.658	**0.896**	-	-	-	-	-	-
EE	0.303	0.440	0.419	0.511	**0.878**	-	-	-	-	-
SI	0.545	0.586	0.502	0.669	0.432	**0.868**	-	-	-	-
FC	0.348	0.505	0.549	0.623	0.628	0.550	**0.875**	-	-	-
BI	0.500	0.638	0.377	0.671	0.508	0.589	0.594	**0.955**	-	-
ADT	0.384	0.416	0.415	0.509	0.422	0.543	0.621	0.563	**0.948**	-
CI	0.510	0.549	0.433	0.656	0.477	0.651	0.608	0.779	0.790	**0.937**

The square root of AVE of each construct (in bold) was larger than its correlation coefficients with other constructs.

**Table A2:** Outer loadings and cross-loadings for the measurement items.

	TAC	TEC	TTF	PE	EE	SI	FC	BI	ADT	CI
TAC1	**0.839**	0.419	0.285	0.445	0.270	0.461	0.283	0.366	0.307	0.398
TAC2	**0.870**	0.474	0.255	0.466	0.256	0.463	0.260	0.405	0.296	0.405
TAC3	**0.870**	0.482	0.214	0.462	0.275	0.446	0.269	0.451	0.285	0.434
TAC4	**0.836**	0.523	0.217	0.473	0.269	0.409	0.302	0.438	0.265	0.407
TAC5	**0.859**	0.517	0.273	0.459	0.268	0.490	0.314	0.413	0.383	0.448
TAC6	**0.879**	0.518	0.226	0.446	0.260	0.491	0.333	0.444	0.371	0.455
TAC7	**0.887**	0.497	0.277	0.438	0.217	0.480	0.318	0.406	0.341	0.446
TAC8	**0.807**	0.553	0.232	0.472	0.267	0.481	0.301	0.526	0.371	0.506
TEC1	0.571	**0.861**	0.401	0.645	0.398	0.512	0.426	0.629	0.404	0.548
TEC2	0.541	**0.903**	0.429	0.628	0.386	0.489	0.466	0.568	0.389	0.492
TEC3	0.514	**0.882**	0.487	0.654	0.379	0.564	0.369	0.560	0.362	0.498
TEC4	0.513	**0.912**	0.497	0.644	0.393	0.527	0.470	0.563	0.380	0.494
TEC5	0.515	**0.900**	0.490	0.626	0.366	0.507	0.467	0.517	0.379	0.472
TEC6	0.468	**0.890**	0.452	0.642	0.422	0.526	0.472	0.556	0.344	0.460
TEC7	0.498	**0.892**	0.437	0.653	0.400	0.528	0.479	0.594	0.342	0.468
TTF1	0.257	0.449	**0.904**	0.551	0.364	0.418	0.495	0.340	0.374	0.367
TTF2	0.284	0.495	**0.926**	0.631	0.406	0.462	0.528	0.408	0.407	0.439
TTF3	0.257	0.479	**0.937**	0.609	0.396	0.483	0.508	0.322	0.383	0.396
TTF4	0.279	0.469	**0.923**	0.634	0.381	0.489	0.494	0.321	0.368	0.393
PE1	0.467	0.592	0.704	**0.885**	0.475	0.611	0.580	0.576	0.481	0.583
PE2	0.502	0.648	0.571	**0.884**	0.414	0.613	0.588	0.537	0.470	0.573
PE3	0.494	0.675	0.530	**0.923**	0.466	0.589	0.526	0.669	0.465	0.631
PE4	0.452	0.668	0.544	**0.893**	0.472	0.587	0.537	0.620	0.407	0.561
EE1	0.313	0.458	0.452	0.533	**0.924**	0.426	0.637	0.503	0.429	0.473
EE2	0.086	0.227	0.219	0.258	**0.779**	0.214	0.395	0.309	0.195	0.252
EE3	0.267	0.393	0.341	0.449	**0.923**	0.362	0.558	0.470	0.342	0.409
EE4	0.335	0.413	0.410	0.490	**0.879**	0.462	0.570	0.460	0.455	0.488
SI1	0.486	0.446	0.404	0.503	0.314	**0.875**	0.415	0.422	0.477	0.531
SI2	0.490	0.470	0.441	0.507	0.325	**0.878**	0.419	0.404	0.458	0.518
SI3	0.450	0.571	0.451	0.676	0.445	**0.852**	0.554	0.635	0.474	0.614
FC1	0.253	0.359	0.468	0.492	0.509	0.447	**0.867**	0.464	0.573	0.508
FC2	0.316	0.397	0.470	0.522	0.603	0.460	**0.897**	0.523	0.575	0.566
FC3	0.340	0.527	0.500	0.608	0.556	0.521	**0.854**	0.559	0.499	0.524
FC4	0.306	0.482	0.481	0.557	0.527	0.498	**0.880**	0.531	0.524	0.526
BI1	0.479	0.615	0.373	0.649	0.507	0.577	0.604	**0.949**	0.531	0.731
BI2	0.480	0.614	0.368	0.658	0.497	0.575	0.582	**0.974**	0.549	0.759
BI3	0.471	0.611	0.334	0.636	0.462	0.530	0.532	**0.950**	0.496	0.718
BI4	0.478	0.596	0.366	0.618	0.470	0.565	0.548	**0.945**	0.571	0.766
ADT1	0.385	0.413	0.399	0.478	0.409	0.528	0.625	0.533	**0.953**	0.740
ADT2	0.374	0.380	0.379	0.442	0.368	0.518	0.548	0.453	**0.917**	0.702
ADT3	0.338	0.390	0.404	0.494	0.407	0.508	0.588	0.554	**0.964**	0.769
ADT4	0.360	0.394	0.393	0.513	0.412	0.507	0.591	0.587	**0.956**	0.782
CI1	0.471	0.511	0.421	0.619	0.453	0.610	0.593	0.740	0.775	**0.956**
CI2	0.512	0.517	0.398	0.614	0.395	0.613	0.540	0.674	0.721	**0.908**
CI3	0.479	0.531	0.420	0.630	0.453	0.625	0.565	0.762	0.755	**0.965**
CI4	0.451	0.500	0.384	0.595	0.488	0.593	0.580	0.743	0.710	**0.920**

The outer loading of each item was higher on its corresponding construct (in bold) than the cross-loadings on other constructs.
